# The effect of acceptance and commitment therapy on negative emotions and quality of life in stroke patients: a systematic review and meta-analysis

**DOI:** 10.3389/fneur.2026.1816395

**Published:** 2026-05-18

**Authors:** Yana Wang, Boling Wei, Lu Wang, Chun Huang, Jifeng Rong, Tianbao Sun, Yu Liu

**Affiliations:** 1 Key Laboratory of Exercise and Health Sciences of Ministry of Education, School of Exercise and Health, Shanghai University of Sport, Shanghai, China; 2 The Center of Rehabilitation Therapy, The First Rehabilitation Hospital of Shanghai, Shanghai, China; 3 Department of Rehabilitation, Shangnan County Hospital, Shangluo, Shaanxi, China; 4 School of Physical Education, Guizhou University of Engineering Science, Bijie, Guizhou, China

**Keywords:** acceptance and commitment therapy, meta-analysis, negative emotions, quality of life, stroke

## Abstract

**Objectives:**

Stroke survivors often experience depression, anxiety and declining quality of life. This systematic review and meta-analysis aimed to evaluate the efficacy of acceptance and commitment therapy (ACT)-based interventions (including ACT alone or ACT combined with conventional rehabilitation) compared with conventional rehabilitation in improving negative emotions and quality of life in stroke patients.

**Methods:**

Two reviewers independently searched PubMed, Web of Science, Embase, the Cochrane Library, CNKI, Wanfang, VIP and SinoMed from database inception to August 31, 2025. Study selection, data extraction, and risk of bias assessment were independently performed by two reviewers. The methodological quality of included randomized controlled trials (RCTs) was evaluated using the Cochrane risk of bias tool. Meta-analyses were conducted using RevMan 5.4. A random-effects model was applied if heterogeneity (I^2^ > 50%) was detected, otherwise a fixed-effects model was used.

**Results:**

Nineteen RCTs involving 1704 patients were included. Meta-analysis showed that ACT significantly alleviated depression (SMD = −1.37, 95% CI −1.86 to −0.87, *p* < 0.00001) and anxiety (SMD = −1.25, 95% CI: −1.76 to −0.74, *p* < 0.00001) compared with control interventions. ACT also significantly enhanced quality of life (SMD = 1.36, 95% CI: 0.69 to 2.02, *p* < 0.0001). Positive effects were also observed on secondary outcomes including psychological flexibility, self-efficacy, sleep quality, and activities of daily living.

**Conclusion:**

ACT is effective in alleviating depression and anxiety, as well as improving quality of life among stroke patients, demonstrating benefits across multiple psychological and functional domains. Future research should focus on large-scale, high-quality RCTs with standardized protocols to confirm long-term efficacy.

**Systematic review registration:**

https://www.crd.york.ac.uk/prospero/, identifier: CRD420251113679.

## 1 Introduction

Stroke is a leading cause of disability and death worldwide (1, 2). Survivors frequently experience substantial physical, cognitive, and emotional disturbances, with depression and anxiety as the most prevalent negative emotional outcomes. The prevalence of post-stroke depression is 33%, and the incidence of anxiety up to 10 years after stroke ranges from 17 to 24% (3, 4). These emotional disorders are not only reactions to physical disability but also act as independent risk factors, hindering rehabilitation participation, slowing functional recovery, and impairing quality of life (QoL). As a multidimensional, patient-centered indicator, QoL has become a core outcome for evaluating holistic post-stroke rehabilitation and is increasingly recognized as a primary therapeutic goal (5). Given the chronic and life-altering nature of post-stroke disability, alleviating depression, anxiety, and compromised QoL in stroke survivors during rehabilitation is an urgent clinical priority.

Conventional psychological interventions, including Positive Psychotherapy (PPT) and Cognitive Behavioral Therapy (CBT), have demonstrated efficacy across a broad range of mental health conditions (6–9). However, they exhibit notable limitations in the stroke population. CBT focuses on identifying and challenging negative automatic thoughts, while PPT prioritizes cultivating positive emotions to counteract negative affect (10). For stroke survivors, the sudden, life-altering nature of stroke, coupled with persistent physical and functional limitations, makes it difficult for patients to fully modify the negative thoughts required by CBT and PPT.

Acceptance and Commitment Therapy (ACT) offers a distinctive approach for stroke survivors, emphasizing psychological flexibility rather than eliminating negative emotions-a core mechanism that directly targets experiential avoidance, a common psychological barrier in post-stroke rehabilitation where patients avoid social interaction or rehabilitation participation to escape distress related to disability (11). ACT can help patients reduce avoidance behaviors, enhance resilience, and rebuild a sense of meaning in life despite physical limitations (12). Emerging evidence has explored the application of ACT in stroke patients, yet findings remain inconsistent and lack critical synthesis. Liu et al. assessed outcomes using health-related quality of life scales and found significant improvements in mental health dimensions in the ACT groups, with no significant differences in physical health dimensions (13). Wang et al. reported that the ACT group exhibited significantly greater improvement in depression compared with the control group, but no significant between-group difference was observed for anxiety (14). Additionally, prior research is hampered by methodological limitations including small single-center sample sizes, heterogeneous ACT intervention protocols, and non-standardized outcome measurements, further contributing to the lack of consensus on ACT’s effectiveness for stroke patients (13–16). To address these critical gaps, this systematic review and meta-analysis aims to comprehensively evaluate the efficacy of ACT for improving depression, anxiety, and QoL in stroke patients, thereby providing evidence-based support for optimizing psychological intervention strategies in clinical stroke rehabilitation practice.

## 2 Materials and methods

This systematic review and meta-analysis was conducted and reported in full accordance with the Preferred Reporting Items for Systematic Reviews and Meta-Analyses (PRISMA) 2020 statement, with no deviations from the guideline requiring additional justification (17). The protocol was registered in PROSPERO (CRD420251113679).

### 2.1 Search strategy

Two reviewers independently searched PubMed, Web of Science, Embase, the Cochrane Library, CNKI, Wanfang, VIP, and SinoMed using a combination of Medical Subject Headings (MeSH) and free-text terms. The search period spanned from database inception to August 31, 2025. References of included studies were also manually screened to identify potentially eligible articles. Searches were restricted to studies published in English or Chinese. Taking the PubMed database as an example, the detailed search strategy is as follows: (“stroke”[Mesh]) AND (“Acceptance and Commitment Therapy”[Mesh] OR “cognitive behavioral therapy” OR “acceptance commitment therapy” OR “acceptance commitment treatment” OR “acceptance commitment” OR “acceptance therapy” OR “acceptance treatment” OR “commitment therapy” OR “commitment treatment” OR “ACT”) AND (“Randomized Controlled Trial”[Publication Type]). The full search strategies for all databases are provided in Appendix 1.

### 2.2 Inclusion and exclusion criteria

#### 2.2.1 Inclusion criteria

Studies were included if they met the following criteria: (1) Participants: Stroke patients aged≥18 years, diagnosed by CT or MRI, without severe cognitive impairment, consciousness disturbances, or speech disorders as defined in the original studies (18). (2) Intervention: acceptance and commitment therapy (ACT), either alone or combined with conventional interventions. (3) Comparison: conventional interventions, including medical treatment, routine nursing, traditional psychotherapy, rehabilitation training, health education, or other standard care. (4) Outcome: primary outcome: depression, anxiety and quality of life; secondary outcome: sleep quality, self-efficacy, psychological flexibility and activities of daily living (ADL). (5) Study design: to ensure high-quality, low risk of bias evidence, only RCTs were included.

#### 2.2.2 Exclusion criteria

Studies were excluded if they were non-RCTs, duplicate publications, studies with incomplete outcome data, reviews, conference abstracts, case reports, or studies for which full text was unavailable.

### 2.3 Literature screening and data extraction

Two reviewers independently performed literature searches to minimize selection bias. Records were imported into EndNote X9 for duplicate removal, which was selected for its robust cross-database duplicate detection and management efficiency. Subsequently, the two reviewers independently screened titles and abstracts, followed by full-text evaluation against predefined inclusion/exclusion criteria. Exclusion reasons were documented. Discrepancies were resolved through discussion, with a third senior reviewer making the final decision if needed. Thereafter, two reviewers independently extracted key data (e.g., first author, year, sample size, age, stroke duration, intervention details, outcome measures). All extracted data were cross-checked, and discrepancies were resolved by consensus. Inter-rater agreement was quantified using Cohen’s kappa (K = 0.88 for screening, K = 0.90 for data extraction), indicating excellent consistency.

### 2.4 Assessment of risk of bias in included studies

Two reviewers independently assessed the risk of bias for all included studies using the Cochrane Collaboration’s Risk of Bias tool (RoB-1) (19). Seven domains were evaluated: random sequence generation, allocation concealment, blinding of participants and personnel, blinding of outcome assessment, incomplete outcome data, selective outcome reporting, and other potential sources of bias. Each domain was judged as low, unclear, or high risk of bias according to standard RoB-1 criteria. Any disagreements between reviewers were resolved by discussion; if no consensus could be reached, a third senior reviewer made the final judgment.

### 2.5 Statistical analysis

Data were organized in Excel and analyzed using RevMan 5.4 software. Continuous outcomes were analyzed using mean and standard deviation of endpoint data. Mean differences (MD) were used for continuous variables with identical measurement tools, and standardized mean differences (SMD) were used for variables assessed with different instruments. Heterogeneity was evaluated using the I^2^ statistic: I^2^ > 50% indicated substantial heterogeneity, in which case random-effects (RE) models were applied, accompanied by sensitivity analyses (leave-one-out) or subgroup analyses. For I^2^ ≤ 50%, fixed-effects (FE) models were used. Prespecified subgroup analyses were performed according to mean age (<60 years vs. ≥60 years), assessment method (Clinician-administered scales vs. Self-reported scales), intervention duration (≤4 weeks vs. >4 weeks), single-session duration (≤30 min vs. >30 min), sample size (≤40 vs. >40), and intervention modality (ACT combined with conventional therapy vs. ACT alone). All tests were two-sided, and *p* < 0.05 was considered statistically significant. Publication bias was assessed for outcomes with 10 or more included studies, using funnel plots visually, supplemented by Egger’s regression and Begg’s tests. *p* > 0.05 was interpreted as indicating no significant publication bias.

## 3 Results

### 3.1 Study selection

**Figure 1:**
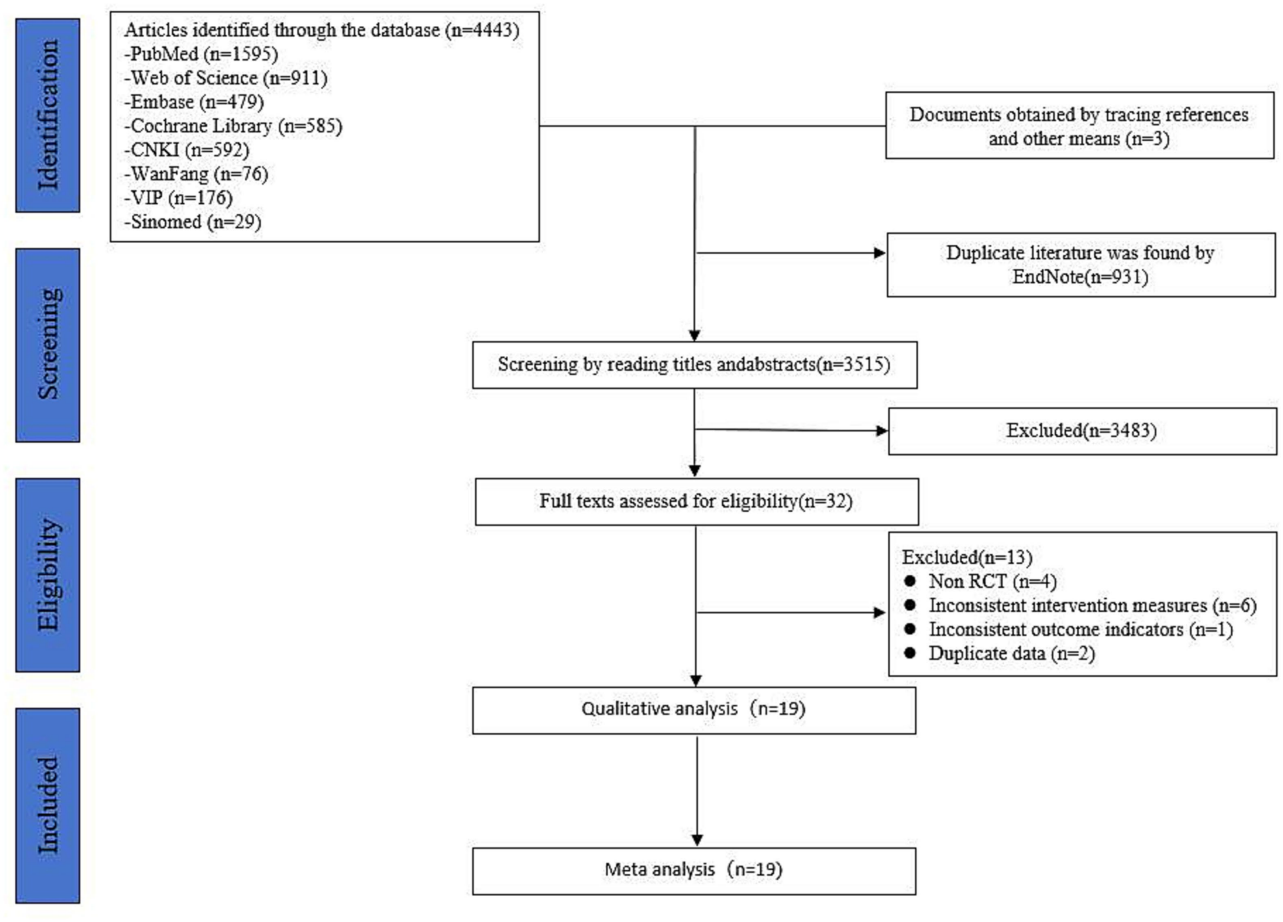
Literature screening process diagram.

A total of 4,446 records were identified through database searching and reference screening. After importing into EndNote and removing 931 duplicates, 3,515 records remained. Following title and abstract screening, 3,483 irrelevant studies were excluded, and 32 studies underwent full-text evaluation. Finally, 19 studies were included in this review. The literature screening process and results are shown in Figure 1.

### 3.2 Research characteristics and risk of bias

The 19 randomized controlled trials included a total of 1704 patients, including 852 in the experimental group and 852 in the control group, published between 2019 and 2025. Among the included studies, four studies were in English and fifteen studies were in Chinese. The intervention measures for the experimental group were acceptance and commitment therapy alone or combined with conventional rehabilitation treatment, while the intervention measures for the control group were conventional rehabilitation treatment, including medical treatment, rehabilitation training, health education, psychological care, psychological guidance, etc. The basic characteristics of the included literature are shown in Table 1.

**Table 1:** Study characteristics of included studies.

Author (year of publication)	Sample size (E/C)	Age, year (E/C)	Duration since stroke onset (E/C)	Intervention (E)	Intervention (C)	Duration of intervention	Tools	Outcomes
Majumdar et al. (2019) (22)	26/27	65.3 ± 11.9/60.0 ± 15.6	14.1 ± 14.5/13.1 ± 13.3	ACT	TAU	2 h/week, 4 weeks	PHQ-9 GAD-7 EQ-5D-5L	①②③
Dai et al. (2020) (24)	54/55	–	–	ACT	Routine psychological care	Once every 2 ~ 3 days, 30 ~ 45 min/session, 5 ~ 6 sessions	AAQ-II	④
Yang et al. (2020) (25)	56/56	55.23 ± 5.84/55.14 ± 5.72	19.28 ± 4.29/19.17 ± 4.34 days	ACT + TAU	TAU (health education and psychological care)	40 min/session, 2 sessions/week, 4 weeks	SCL-90 GQOLI-74	①③
Shi et al. (2021) (26)	40/41	63.32 ± 1.17/62.51 ± 1.23	15 ± 1.9/14 ± 2.1 days	ACT + TAU	TAU (routine treatment, rehabilitation training, health education, psychological guidance, etc.)	2 sessions/week, 20 ~ 30 min/session, 4 weeks	GDS-15 GAD-7 GSES	①②⑤
Niu et al. (2022) (23)	52/52	61.5 ± 11.5/64.8 ± 12.1	<1 week	ACT + Usual medical care	Usual medical care	45 ~ 55 min/session, 5 sessions, 2 weeks	HAMD BI	①⑥
Wang et al. (2022) (27)	50/50	62.04/60.86	–	ACT	Traditional psychological nursing	1 month	SDS SAS AAQ-II PSQI	①②④⑦
Hao et al. (2022) (28)	62/60	43.5 ± 3.25/44.1 ± 4.16	20.8 ± 2.57/21.2 ± 3.22 days	ACT + CT	Routine health education and psychological care	2 sessions/week, 15 min/session	SDS SAS SWLS KPS ADL	①②③⑥
Li et al. (2022) (29)	60/60	60.47 ± 7.13/61.22 ± 6.89	–	ACT + TAU	TAU	Once every 1 ~ 2 days, 15–20 min/session, 7 days	GSES	⑤
Liu (2022) (30)	32/33	18 ~ 75/18 ~ 76	–	ACT	Traditional psychological nursing	45 ~ 60 min/session, 7 sessions, 6 weeks	PHQ-9 GAD-7	①②
Liu et al. (2022) (31)	45/45	59.22 ± 10.27/59.04 ± 10.35	–	ACT + TAU	TAU	1 h/session, 2 sessions/week, 4 weeks	MSSNS GQOLI-74	①②③
Liu et al. (2023) (13)	70/69	62.21 ± 9.937/61.61 ± 10.84	≤2 weeks	ACT + TAU	TAU	45 ~ 60 min/session, 7 sessions, 4 weeks	HAMD MCS PCS AAQ-II PSQI	①③④⑦
Huang (2023) (32)	34/34	61.25 ± 3.53/62.38 ± 4.07	–	ACT + TAU	TAU	1 session/2 weeks, 4 week	SDS SAS GSES BI	①②⑤⑥
Jia et al. (2023) (33)	30/30	59.70 ± 11.38/62.07 ± 10.43	–	ACT	TAU	30 ~ 40 min/session, 2 weeks	HADS-D HADS-A AAQ-II PSQI	①②④⑦
Wang et al. (2023) (34)	40/40	64.12 ± 2.47/63.95 ± 2.51	–	ACT + TAU	TAU	30 min/session, 3 sessions/week, 3 weeks	SV-SS-QoL	③
Yang (2023) (35)	35/35	63.60 ± 7.66/62.20 ± 6.17	12.45 ± 5.97/11.55 ± 5.31 weeks	ACT + Basic treatment	Basic treatment	40 min/session, 1 session/2 days, 4 weeks	HAMD HAMA MBI	①②⑥
Yang et al. (2023) (36)	45/45	–	–	ACT + Medical treatment	Medical treatment	40 min/session, 2 sessions/week, 16 sessions	HAMD HAMA	①②
Wang et al. (2024) (14)	46/45	52.04 ± 7.93/51.86 ± 7.81	18.23 ± 6.85/17.81 ± 6.73 days	ACT + TAU	TAU	40 ~ 60 min/session, 1 session/week, 7 weeks	SDS SAS	①②
Cheng (2024) (15)	35/35	59.80 ± 5.54/60.31 ± 5.73	–	ACT + Health education guidance	Health education guidance	7 sessions, 4 weeks	AAQ-II SS-QOL	③④
Ding (2025) (16)	40/40	66.22 ± 4.79/65.17 ± 5.05	–	ACT + Early rehabilitation nursing	Early rehabilitation nursing	1 session/3 days, 30 min/session	HAMD HAMA	①②

ACT, acceptance and commitment therapy; TAU, treatment as usual. ① Depression: PHQ-9, The Patient Health Questionnaire-9; SCL-90, Symptom checklist 90; GDS-15, Geriatric Depression Scale-15; HAMD, 24-item Hamilton Depression Scale; SDS, Self-Rating Depression Scale; MSSNS, Mental Status Scale in Non-psychiatric Settings; HADS-D, Hospital Anxiety and Depression Scale Depression subscale; ② Anxiety: Hamilton anxiety scale, HAMA; GAD-7, The Generalized Anxiety Disorder-7; SAS, Self-Rating Anxiety Scale; MSSNS, Mental Status Scale in Non-psychiatric Settings; HADS-A, Hospital Anxiety and Depression Scale Anxiety subscale; ③ Quality of life: EQ-5D-5L, EuroQol five-dimensional questionnaire with five levels; GQOLI-74, Generic Quality of Life Inventory; SWLS, satisfaction with life scale; MCS, mental component summary section in Short Form Survey Version 2; PCS, Physical component summary section in Short Form Survey Version 2; SV-SS-QoL, the Short Version of the stroke specific quality of life scale; SS-QOL, Stroke-Specific Quality of Life Scale; ④ Psychological flexibility: AAQ-II, Acceptance and Action Questionnaire-II; ⑤ Self-Efficacy: GSES, General Self-Efficacy Scale-Schwarzer; ⑥ Activities of Daily Living: BI, Barthel Index; KPS, Karnofsky performance score; ADL, Activities of Daily Living Scale; ⑦ Sleep quality: PSQI, Pittsburgh sleep quality index.

**Figure 2:**
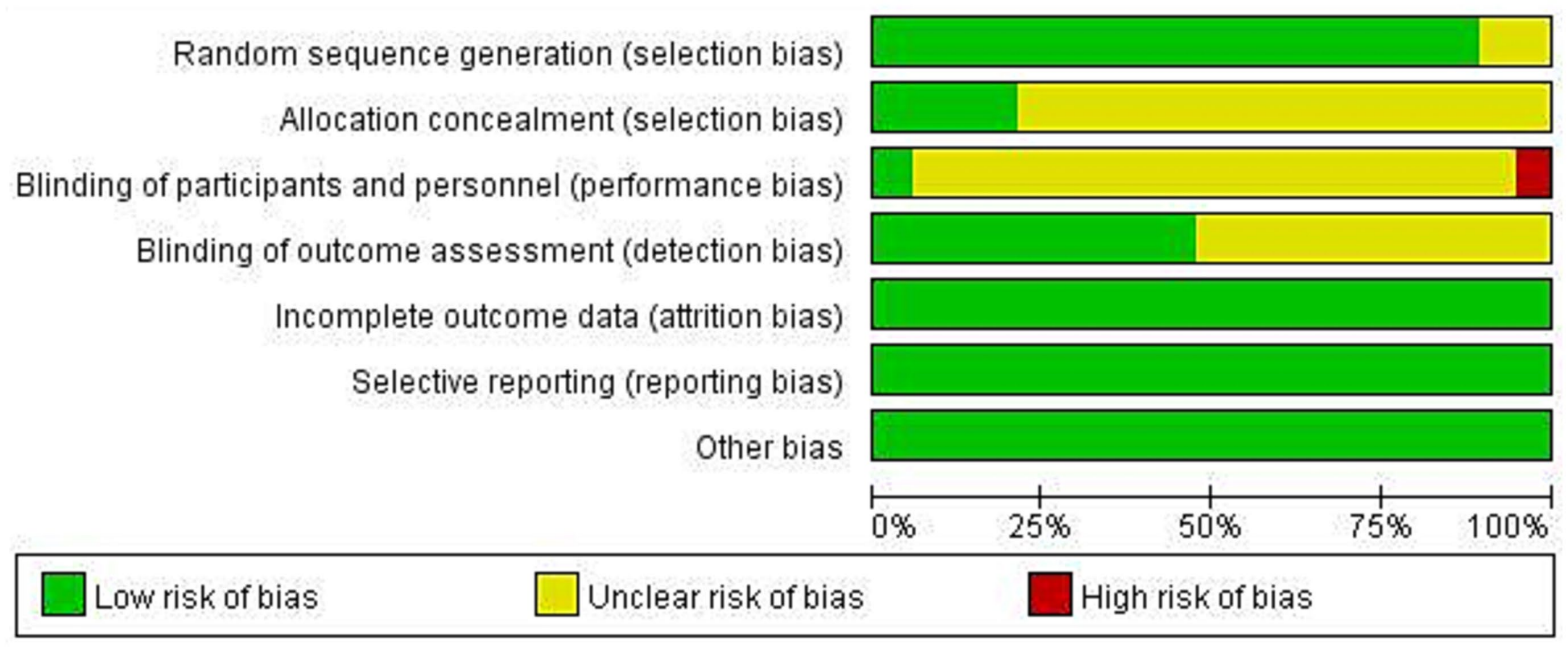
Risk of bias graph.

**Figure 3:**
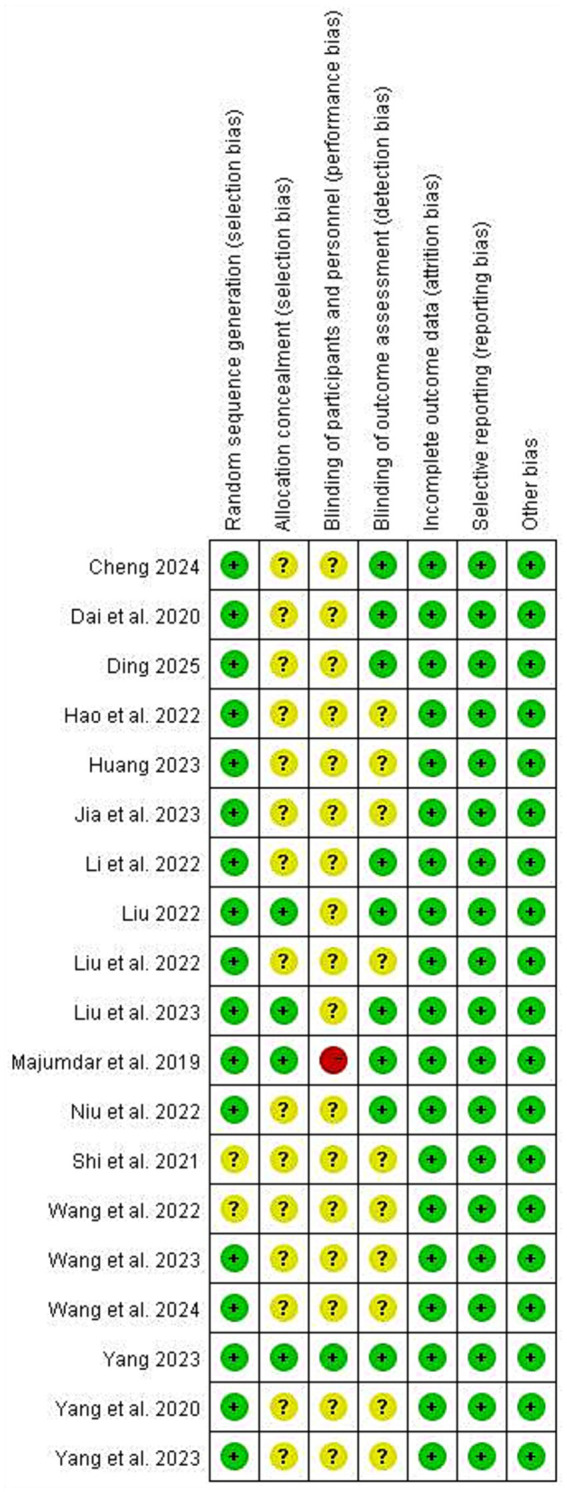
Risk of bias summary.

Two studies only reported random allocation and did not report specific methods. Fifteen studies did not indicate allocation concealment and were considered to have an unclear risk of bias. Regarding blinding of participants and personnel, except for one study that was double-blinded, one study rated as high risk clearly stated that blinding of participants was not possible during the intervention process, and the rest of the studies did not mention blinding. For blinding of outcome assessors, only nine studies mentioned relevant information. In addition, no other bias risks were found. The overall quality evaluation results of the included literature are shown in Figures 2, 3.

### 3.3 Meta-analysis

#### 3.3.1 Depression

**Figure 4:**
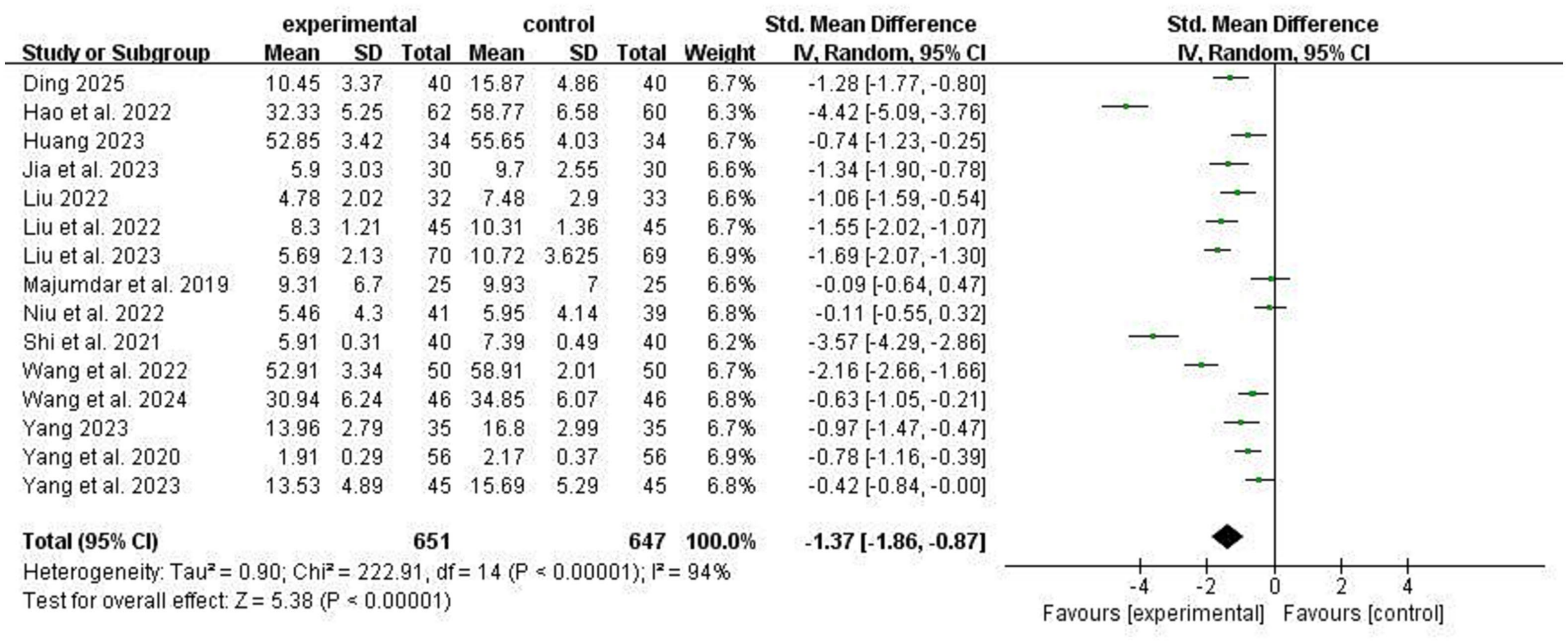
Comparison of depression improvement between experimental group and control group.

Fifteen studies reported the effects of ACT on depressive symptoms in stroke patients. High heterogeneity was detected across studies (I^2^ = 94%); thus, a random-effects model was adopted. The results showed that, compared with the conventional group, ACT significantly alleviated depressive symptoms in stroke patients [SMD = −1.37, 95% CI − 1.86 to −0.87, *p* < 0.00001], as illustrated in Figure 4. Subgroup analyses were conducted based on mean age, assessment method, intervention duration, single-session duration, and sample size. ACT remained effective across all subgroups compared with the conventional group, but there were no significant between-subgroup differences (*P* > 0.05; Table 2). Notably, as shown in Table 2, ACT combined with conventional rehabilitation showed a marginally significant greater improvement in depressive symptoms compared with ACT alone (*p* = 0.05).

**Table 2:** Subgroup analysis.

Outcome or subgroup	Studies	Participants	I^2^%	Effect estimate	*p*
Depression					
Age (year)					0.22
<60 years	5	476	96	−1.72 [−2.81, −0.63]	0.002
≥60 years	8	667	93	−1.31 [−1.99, −0.63]	0.0002
Not given	2	155	72	−0.72 [−1.35, −0.09]	0.02
Assessment method					0.19
Clinician-rating scales	5	481	87	−0.97 [−1.50, −0.43]	0.0004
Self-rating scales	10	817	95	−1.58 [−2.32, −0.84]	<0.0001
Invention modality					0.05
ACT alone	4	267	74	−0.78 [−1.27, −0.28]	0.002
ACT + Conventional rehabilitation	11	1,031	95	−1.58 [−2.22, −0.94]	<0.00001
Intervention duration (week)					0.08
≤4	10	849	92	−1.28 [−1.82, −0.74]	<0.00001
>4	3	247	44	−0.68 [−1.02, −0.33]	0.0001
Not given	2	202	98	−2.84 [−5.92, 0.23]	0.07
Single-session duration (minute)					0.12
≤30	3	282	97	−3.08 [−5.10, −1.06]	0.003
>30	9	798	82	−0.94 [−1.30, −0.59]	<0.00001
Not given	3	218	94	−1.00 [−2.19, 0.19]	0.10
Sample size					0.35
≤40	7	473	90	−1.27 [−1.92, −0.62]	0.0001
>40	8	1,037	96	−1.75 [−2.52, −0.98]	<0.0001
Anxiety					
Age (year)					0.42
<60 years	5	475	96	−1.44 [−2.47, −0.42]	0.006
≥60 years	6	447	92	−1.26 [−2.02, −0.51]	0.001
Not given	2	155	74	−0.73 [−1.39, −0.08]	0.03
Assessment method					0.51
Clinician-administered scales	3	240	89	−0.98 [−1.80, −0.15]	0.02
Self-reported scales	10	837	94	−1.33 [−1.97, −0.70]	<0.0001
Intervention modality					0.97
ACT alone	4	273	93	−1.23 [−2.28, −0.18]	0.02
ACT + other therapy	9	804	94	−1.26 [−1.87, −0.64]	<0.0001
Intervention duration (week)					0.05
≤4	8	629	88	−1.09 [−1.59, −0.58]	<0.0001
>4	3	246	62	−0.60 [−1.01, −0.18]	0.005
Not given	2	202	96	−2.88 [−4.91, −0.85]	0.005
Single-session duration (minute)					0.24
≤30	3	283	97	−2.14 [−3.94, −0.33]	0.02
>30	7	578	49	−0.77 [−1.01, −0.53]	<0.00001
Not given	3	216	96	−1.46 [−2.99, 0.07]	0.06
Sample size					0.38
≤40	7	472	82	−1.03 [−1.49, −0.57]	<0.0001
>40	6	605	96	−1.51 [−2.49, −0.54]	0.002
Quality of life					
Age (year)					0.009
<60 years	4	394	92	2.07 [1.18, 2.95]	<0.00001
≥60 years	3	406	86	0.66 [0.09, 1.23]	0.02
Single-session duration (minute)					0.49
≤30	2	202	88	1.60 [0.66, 2.54]	0.0009
>30	3	480	97	1.55 [0.39, 2.72]	0.009
Not given	2	118	89	0.73 [−0.44, 1.91]	0.22
Sample size					0.20
≤40	3	198	81	0.87 [0.18, 1.55]	0.01
>40	4	602	96	1.65 [0.68, 2.63]	0.0009

#### 3.3.2 Anxiety

**Figure 5:**
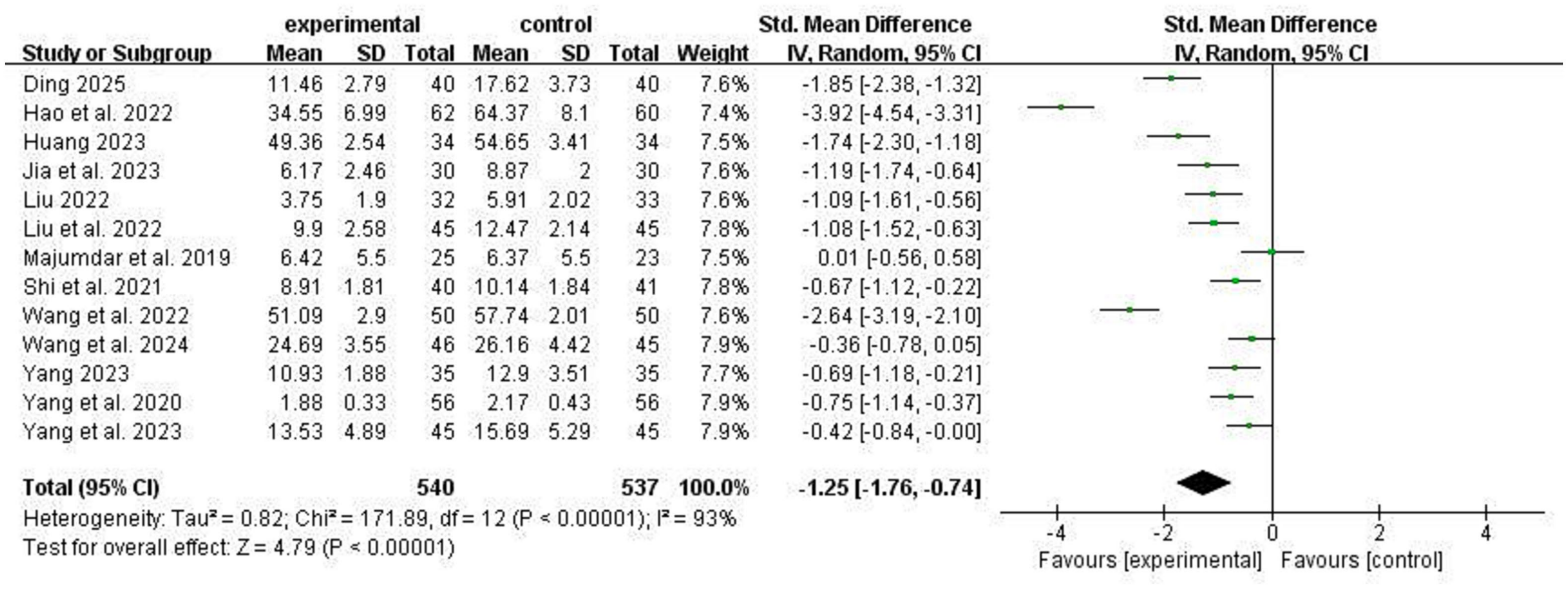
Comparison of anxiety improvement between experimental group and control group.

Thirteen studies reported the effects of ACT on anxiety symptoms in stroke patients. High heterogeneity was detected across studies (I^2^ = 93%); thus, a random-effects model was employed. Compared with conventional treatment, ACT significantly alleviated anxiety in stroke patients [SMD = −1.25, 95% CI − 1.76 to −0.74, *p* < 0.00001], as illustrated in Figure 5. Subgroup analyses were conducted according to mean age, assessment method, intervention modality, single-session duration, and sample size. ACT remained effective across all subgroups relative to the conventional group, with no statistically significant between-subgroup differences (*P* > 0.05; Table 2). Notably, as shown in Table 2, ACT intervention of 4 weeks or shorter showed a marginally significant greater improvement in anxiety symptoms compared with interventions longer than 4 weeks (*p* = 0.05).

#### 3.3.3 Quality of life

**Figure 6:**
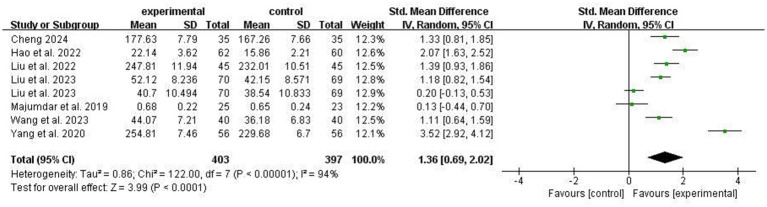
Comparison of quality of life improvement between experimental group and control group.

Seven studies reported the effect of ACT on quality of life in stroke patients. High heterogeneity was observed across studies (I^2^ = 94%); thus, a random-effects model was applied. Pooled results demonstrated a statistically significant between-group difference in quality of life improvement favoring the ACT group over the control group [SMD = 1.36, 95% CI 0.69 to 2.02, *p* < 0.0001] (Figure 6). Subgroup analyses were performed according to single-session duration and sample size. ACT remained effective for improving quality of life across all subgroups relative to conventional treatment, with no significant between-subgroup differences (*p* > 0.05, Table 2). Notably, as shown in Table 2, a significantly larger effect size of ACT on quality of life was observed in patients aged <60 years (*p* = 0.009).

#### 3.3.4 Sleep quality

**Figure 7:**
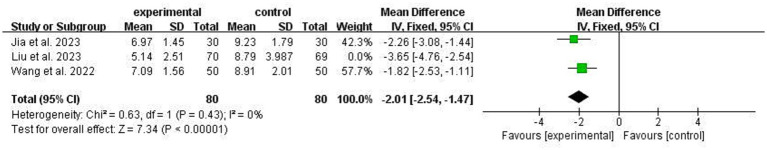
Comparison of sleep quality improvement between experimental group and control group.

Three studies reported the impact of ACT on sleep quality in stroke patients using the Pittsburgh sleep quality index scale, with high heterogeneity between studies (I^2^ = 73%). Sensitivity analysis was conducted using a one by one exclusion method, and after removing Liu et al.’s study, heterogeneity decreased, with I^2^ = 0 (Figure 7). The results showed that ACT can improve the sleep quality of stroke patients, with statistical differences compared to the control group [MD = −2.01, 95% CI −2.54 to −1.47, *p* < 0.00001].

#### 3.3.5 Self-efficacy

**Figure 8:**
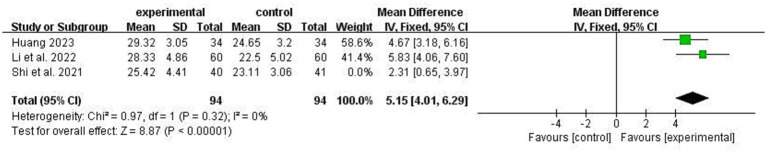
Comparison of self-efficacy improvement between experimental group and control group.

Three studies reported the impact of ACT on self-efficacy in stroke patients using the General Self-Efficacy Scale-Schwarzer scale, with high heterogeneity between studies (I^2^ = 77%). Sensitivity analysis was conducted using a one by one exclusion method, and after removing one study, I^2^ = 0 (Figure 8). The results showed that ACT could improve the self-efficacy of stroke patients, and the difference between the two groups was statistically significant [MD = 5.15, 95% CI 4.01 to 6.29, *p* < 0.00001].

#### 3.3.6 Psychological flexibility

**Figure 9:**
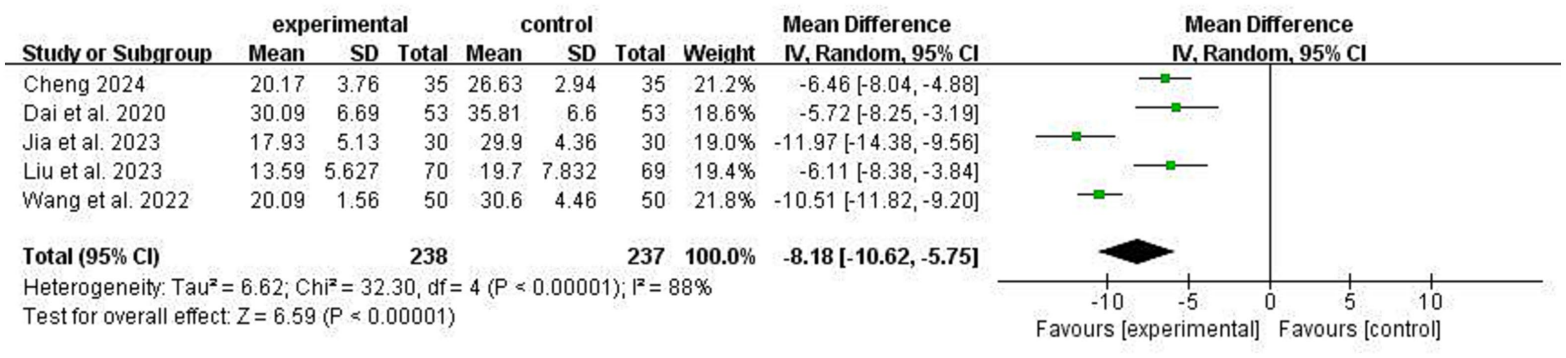
Comparison of psychological flexibility improvement between experimental group and control group.

Five studies reported the effect of ACT on psychological flexibility in stroke patients using the Acceptance and Action Questionnaire-II scale, with high heterogeneity between studies (I^2^ = 88%). Sensitivity analysis revealed that I^2^ = 0 after excluding Jia et al. and Wang et al. (Figure 9). The results showed that ACT could improve the psychological flexibility of stroke patients, and the difference between the two groups was statistically significant [MD = −8.18, 95% CI −10.62 to −5.75, *p* < 0.00001].

#### 3.3.7 ADL

**Figure 10:**
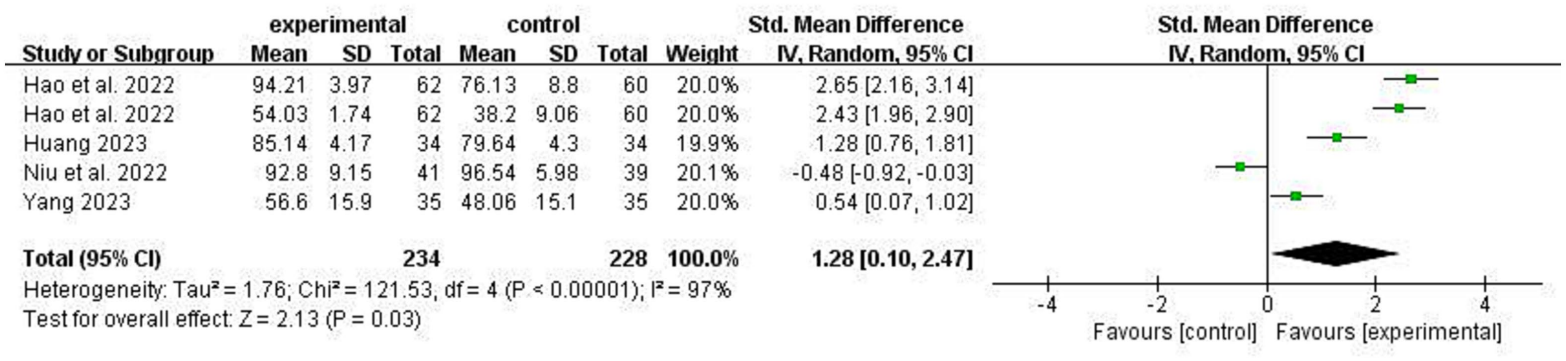
Comparison of ADL improvement between experimental group and control group.

Four studies investigated the impact of ACT on daily living activities in stroke patients. Among them, Hao et al. used Karnofsky performance score and ADL scales for evaluation. The heterogeneity between studies was high (I^2^ = 97%), so a random effects model was used. The results showed that there was a statistically significant difference in ADL scores between the experimental group and the control group [SMD = 1.28, 95% CI 0.10 to 2.47, *p* = 0.03], as shown in Figure 10.

#### 3.3.8 Publication bias

**Figure 11:**
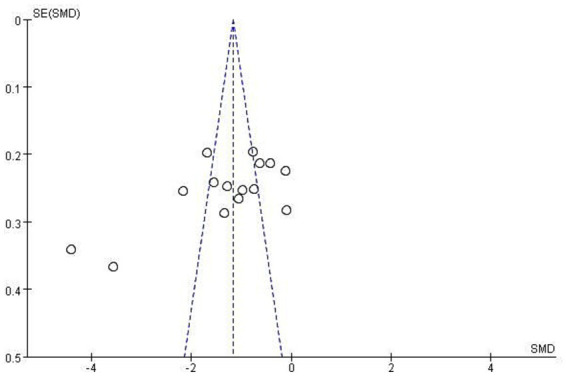
Funnel plot of depression.

**Figure 12:**
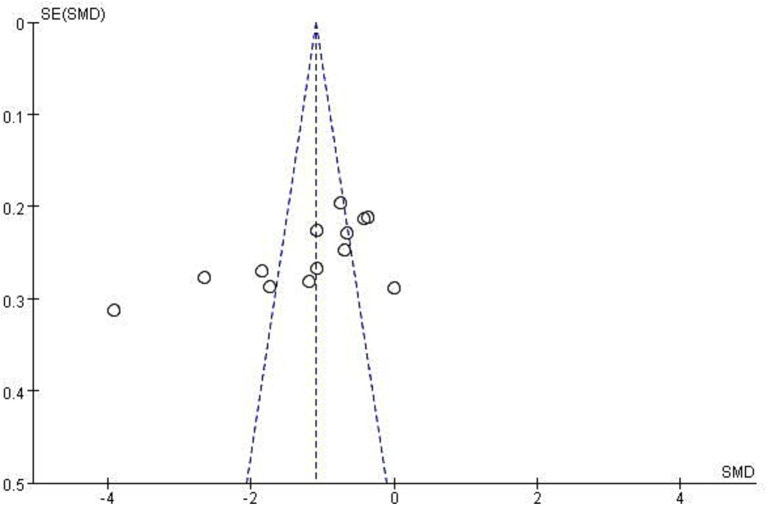
Funnel plot of anxiety.

Funnel plots displayed mild asymmetry for both depression and anxiety outcomes (Figures 11, 12). Begg’s test (depression: *p* = 0.11; anxiety: *p* < 0.05) and Egger’s test (depression: *p* < 0.05; anxiety: *p* < 0.05) suggested potential publication bias. The trim-and-fill method was applied for correction, yielding adjusted pooled effect sizes for depression [SMD = −1.677, 95% CI − 2.220 to −1.134] and anxiety [SMD = −1.458, 95% CI − 1.968 to −0.930], supporting the robustness of the main findings.

## 4 Discussion

This systematic review and meta-analysis synthesized evidence from 19 randomized controlled trials involving 1704 stroke patients to comprehensively evaluate the efficacy of ACT either as monotherapy or in combination with conventional rehabilitation for improving negative emotions, quality of life, and a panel of secondary psychosocial and functional outcomes. Overall, our findings confirmed that ACT-based intervention exerted a significant positive effect on stroke patients compared with conventional rehabilitation alone, which markedly alleviated depressive and anxiety symptoms, enhanced overall quality of life, and also showed favorable impacts on psychological flexibility, self-efficacy, sleep quality, and activities of daily living.

Our core findings align with the limited existing evidence on ACT for stroke patients reported by Zhang et al. (20), which also verified the positive effects of ACT on negative emotions and quality of life. Beyond replicating these prior conclusions, the present study extends the evidence base by providing pooled quantitative data on under-investigated secondary outcomes, including sleep quality, psychological flexibility, and ADL. This expansion is clinically meaningful, as it demonstrates that ACT’s benefits are not limited to emotional regulation but also extend to key domains that directly influence stroke patients’ daily functioning and rehabilitation progress.

Given the substantial statistical heterogeneity observed across nearly all outcome measures, we conducted leave-one-out sensitivity analyses to test the robustness of our pooled estimates. Visual inspection of forest plots also identified several potential outlier studies characterized by unusually large effect sizes or wide confidence intervals, raising initial concerns about their impact on pooled results. Nevertheless, sensitivity analyses revealed that omitting any single study, including these potential outliers, had minimal impact on the primary outcomes, and core conclusions remained unchanged. These results collectively indicate that our meta-analytic findings are stable, reliable, and not unduly influenced by any individual trial.

Subgroup analyses further explored the potential moderating effects of clinical and methodological factors on ACT’s efficacy. For depressive and anxiety symptoms, factors including patient age, assessment method, intervention duration, single-session duration, and sample size did not yield statistically significant between-subgroup differences. This consistency highlights that ACT has stable therapeutic effects across diverse clinical scenarios and patient characteristics, supporting its broad applicability in stroke rehabilitation. Marginally significant differences were only observed for intervention modality in depression outcomes and total intervention duration in anxiety outcomes: ACT combined with conventional rehabilitation appeared to have a slightly stronger effect on depressive symptom relief, and shorter intervention regimens were associated with more rapid anxiety reduction. However, these findings were at the statistical borderline, and their clinical relevance remains to be confirmed by larger, well-standardized trials with sufficient statistical power. For quality of life, age emerged as a key moderating factor, with significantly more pronounced benefits observed in patients under 60 years of age. This age-related difference is likely attributable to the higher prevalence of comorbid chronic diseases, greater baseline functional decline, and reduced psychological and cognitive adaptability in older stroke patients (21), which may attenuate the psychosocial benefits of ACT. Notably, several subgroup analyses were limited by the small number of included studies, and a subset of trials failed to report stroke duration data, precluding further subgroup exploration by disease chronicity, thus, interpretations of these subgroup results should be made with caution.

Substantial between-study heterogeneity was observed across analyses. This variability may stem from multiple sources, including inconsistent outcome measurement tools, heterogeneous ACT intervention protocols, differences in patient baseline characteristics, and variations in methodological quality across included trials. As subgroup analyses did not identify a single dominant moderator, heterogeneity likely reflects the combined influence of these overlapping clinical and methodological factors. Although high heterogeneity limits definitive conclusions regarding an optimal ACT regimen, the stability observed in sensitivity analyses supports the robustness of our overall findings.

Publication bias was assessed for depressive and anxiety symptoms. Evidence of asymmetry was detected, indicating the presence of publication bias, a frequent limitation in meta-analyses of psychological interventions often attributed to the underrepresentation of null or negative findings. Nevertheless, trim-and-fill correction did not materially alter the significance or direction of pooled estimates, confirming that our core conclusions regarding the efficacy of ACT remain robust despite this bias. These findings highlight the importance of prospective trial registration and more complete reporting of non-significant results in future stroke rehabilitation research.

Notably, a small number of individual studies reported non-significant between-group differences that were inconsistent with our overall meta-analytic conclusions. For primary outcomes, two trials found no significant ACT effect on depression (22, 23), and another two found no effect on anxiety (14, 22); two trials also reported no difference in quality of life between the ACT and control groups (13, 14). Several factors may explain these discrepant findings. First, analytical strategy differences between the present meta-analysis and individual trials: most original studies used pre-intervention to post-intervention change scores for group comparisons, a more sensitive approach for capturing intervention effects, whereas our analysis relied on post-intervention endpoint values alone. Although baseline data were statistically comparable across groups in included trials, minor baseline imbalances may have reduced the ability of endpoint-only analyses to detect true intervention effects, masking small but meaningful benefits in some studies. Second, ACT’s core therapeutic mechanism: ACT targets psychological flexibility and the acceptance of disease-related distress, rather than direct physical function improvement-this explains why one trial found no significant ACT effect on the physical component summary of quality of life, even as the mental component summary improved significantly. Third, intervention duration limitations: one trial reporting no ACT effect on ADL used an extremely short intervention period (only 2 weeks), and behavioral and functional changes induced by ACT require sustained practice and cumulative exposure (23); short-term interventions are therefore unlikely to yield measurable functional benefits. Fourth, methodological limitations of individual trials: small sample sizes, insufficient statistical power, and low sensitivity of some assessment tools to detect subtle clinical changes may also contribute to null findings in individual studies. For secondary outcomes, the limited number of included trials further reduces the stability of our pooled estimates for these endpoints, and additional high-quality research is needed to confirm ACT’s effects on sleep quality, self-efficacy, and ADL in stroke patients.

This study has several important limitations that must be acknowledged when interpreting the findings. First, methodological quality variability among included trials: a subset of studies lacked clear and transparent reporting of key methodological details, including random sequence generation, allocation concealment, and reasons for missing data, introducing a potential risk of bias. Second, a paucity of high-quality trials: the included studies were predominantly small, single-center RCTs, with no large, multicenter trials identified, this limits the generalizability of our findings to broader clinical populations. Third, substantial statistical heterogeneity and confirmed publication bias may affect the precision of our pooled effect size estimates, despite the robustness of our core conclusions. Fourth, short follow-up periods, most included trials only assessed outcomes immediately post-intervention, with no long-term follow-up data available, precluding an evaluation of the sustainability of ACT’s effects in stroke patients.

These limitations highlight clear directions for future research to advance the evidence base for ACT in stroke rehabilitation. First, consensus on core outcome measures is urgently needed: the field should adopt standardized, validated scales for key outcomes (e.g., PHQ-9 for depression, GAD-7 for anxiety, Barthel Index for ADL) to reduce measurement heterogeneity and facilitate cross-study comparison. Second, development of a standardized ACT intervention protocol for stroke patients is essential, including clear definitions of total duration (we recommend a minimum of 8 weeks to ensure sufficient exposure), session length, delivery format, and integration with conventional rehabilitation; this will enable definitive comparisons of ACT’s efficacy across trials and identification of the optimal implementation strategy. Third, large, multicenter, adequately blinded RCTs are required, particularly for highly heterogeneous outcomes such as anxiety and ADL, to increase statistical power, improve generalizability, and address the paucity of high-quality evidence. Fourth, long-term follow-up assessments should be incorporated into future trials to evaluate the durability of ACT’s effects on emotional and functional outcomes in stroke patients, a critical consideration for clinical rehabilitation practice. Finally, greater emphasis on publishing negative or inconclusive results and prospective trial registration will help reduce publication bias and improve the completeness of future meta-analyses.

## 5 Conclusion

ACT can significantly improve the negative emotions of depression and anxiety in stroke patients and enhance their quality of life. It has shown positive intervention effects in psychological flexibility, self-efficacy, sleep quality, and daily living activities. At present, research on ACT in the field of stroke rehabilitation is still scarce. In the future, large-scale, high-quality randomized controlled trials need to be conducted, and attention should be paid to its long-term efficacy and cost-effectiveness, in order to provide more reliable evidence support for clinical practice.

## Data Availability

The original contributions presented in the study are included in the article/Supplementary material, further inquiries can be directed to the corresponding author.
