# Supplementary material for: The effect of acceptance and commitment therapy on negative emotions and quality of life in stroke patients: a systematic review and meta-analysis
Source: Front Neurol. 2026 May 18;17:1816395. doi: 10.3389/fneur.2026.1816395 (PMC13224744; doi:10.3389/fneur.2026.1816395)
Supplement: Supplementary file 1 [file Table_1.docx]

Appendix 1 Search strategy

| Database | Search strategy | |
| --- | --- | --- |
| PubMed | #1 | "stroke"[Mesh] OR “Strokes” OR “cerebrovascular accident” OR “cerebrovascular accidents” OR “CVA” OR “CVAs” OR “cerebrovascular apoplexy” OR “apoplexy, cerebrovascular” OR “vascular accident, brain” OR “brain vascular accident” OR “brain vascular accidents” OR “vascular accidents, brain” OR “cerebrovascular stroke” OR “cerebrovascular strokes” OR “stroke, cerebrovascular” OR “strokes, cerebrovascular” OR “apoplexy” OR “cerebral stroke” OR “cerebral strokes” OR “stroke, cerebral” OR “strokes, cerebral” OR “stroke, acute” OR “acute stroke” OR “acute strokes” OR “strokes, acute” OR “cerebrovascular accident, acute” OR “acute cerebrovascular accident” OR “acute cerebrovascular accidents” OR “cerebrovascular accidents, acute” |
|  | #2 | "Acceptance and Commitment Therapy"[Mesh] OR “cognitive behavioral therapy” OR “acceptance commitment therapy” OR “acceptance commitment treatment” OR “acceptance commitment” OR “acceptance therapy” OR “acceptance treatment” OR “commitment therapy” OR “commitment treatment” OR “ACT” |
|  | #3 | "Randomized Controlled Trial"[Publication Type] OR randomized[Title/Abstract] OR placebo[Title/Abstract] OR “RCT” |
|  | #4 | #1 and #2 and #3 |
| Web of Science | #1 | TS=(stroke OR Strokes OR cerebrovascular accident OR cerebrovascular accidents OR CVA OR CVAs OR cerebrovascular apoplexy OR apoplexy, cerebrovascular OR vascular accident, brain OR brain vascular accident OR brain vascular accidents OR vascular accidents, brain OR cerebrovascular stroke OR cerebrovascular strokes OR stroke, cerebrovascular OR strokes, cerebrovascular OR apoplexy OR cerebral stroke OR cerebral strokes OR stroke, cerebral OR strokes, cerebral OR stroke, acute OR acute stroke OR acute strokes OR strokes, acute OR cerebrovascular accident, acute OR acute cerebrovascular accident OR acute cerebrovascular accidents OR cerebrovascular accidents, acute) |
|  | #2 | TS=(Acceptance and Commitment Therapy OR cognitive behavioral therapy OR acceptance commitment therapy OR acceptance commitment treatment OR acceptance commitment OR acceptance therapy OR acceptance treatment OR commitment therapy OR commitment treatment OR ACT) |
|  | #3 | TS=(Randomized Controlled Trial OR randomized OR placebo OR RCT) |
|  | #4 | #1 and #2 and #3 |
| Cochrane Library | #1 | MeSH descriptor: [Stroke] explode all trees |
|  | #2 | (Strokes):ab,kw,ti OR (Cerebrovascular Accident):ab,kw,ti OR (Cerebrovascular Accidents):ab,kw,ti OR (CVA):ab,kw,ti OR (CVAs):ab,kw,ti OR (Cerebrovascular Apoplexy):ab,kw,ti OR (Apoplexy, Cerebrovascular):ab,kw,ti OR (Vascular Accident, Brain):ab,kw,ti OR (Brain Vascular Accident):ab,kw,ti OR (Brain Vascular Accidents):ab,kw,ti OR (Vascular Accidents, Brain):ab,kw,ti OR (Cerebrovascular Stroke):ab,kw,ti OR (Cerebrovascular Strokes):ab,kw,ti OR (Stroke, Cerebrovascular):ab,kw,ti OR (Strokes, Cerebrovascular):ab,kw,ti OR (Apoplexy):ab,kw,ti OR (Cerebral Stroke):ab,kw,ti OR (Cerebral Strokes):ab,kw,ti OR (Stroke, Cerebral):ab,kw,ti OR (Strokes, Cerebral):ab,kw,ti OR (Stroke, Acute):ab,kw,ti OR (Acute Stroke):ab,kw,ti OR (Acute Strokes):ab,kw,ti OR (Strokes, Acute):ab,kw,ti OR (Cerebrovascular Accident, Acute):ab,kw,ti OR (Acute Cerebrovascular Accident):ab,kw,ti OR (Acute Cerebrovascular Accidents):ab,kw,ti OR (Cerebrovascular Accidents, Acute):ab,kw,ti |
|  | #3 | #1 OR #2 |
|  | #4 | MeSH descriptor: [Acceptance and Commitment Therapy] explode all trees |
|  | #5 | (cognitive behavioral therapy):ab,kw,ti OR (acceptance commitment therapy):ab,kw,ti OR (acceptance commitment treatment):ab,kw,ti OR (acceptance commitment):ab,kw,ti OR (acceptance therapy):ab,kw,ti OR (acceptance treatment):ab,kw,ti OR (commitment therapy):ab,kw,ti OR (commitment treatment):ab,kw,ti OR (ACT):ab,kw,ti |
|  | #6 | #4 OR #5 |
|  | #7 | (Randomized Controlled Trial):ab,kw,ti OR (randomized):ab,kw,ti OR (placebo):ab,kw,ti OR (RCT):ab,kw,ti |
|  | #8 | #3 AND #6 AND #7 |
| Embase | #1 | ‘Stroke’:ab,kw,ti OR ‘Strokes’:ab,kw,ti OR ‘Cerebrovascular Accident’:ab,kw,ti OR ‘Cerebrovascular Accidents’:ab,kw,ti OR ‘CVA’:ab,kw,ti OR ‘CVAs’:ab,kw,ti OR ‘Cerebrovascular Apoplexy’:ab,kw,ti OR ‘Apoplexy, Cerebrovascular’:ab,kw,ti OR ‘Vascular Accident, Brain’:ab,kw,ti OR ‘Brain Vascular Accident’:ab,kw,ti OR ‘Brain Vascular Accidents’:ab,kw,ti OR ‘Vascular Accidents, Brain’:ab,kw,ti OR ‘Cerebrovascular Stroke’:ab,kw,ti OR ‘Cerebrovascular Strokes’:ab,kw,ti OR ‘Stroke, Cerebrovascular’:ab,kw,ti OR ‘Strokes, Cerebrovascular’:ab,kw,ti OR ‘Apoplexy’:ab,kw,ti OR ‘Cerebral Stroke’:ab,kw,ti OR ‘Cerebral Strokes’:ab,kw,ti OR ‘Stroke, Cerebral’:ab,kw,ti OR ‘Strokes, Cerebral’:ab,kw,ti OR ‘Stroke, Acute’:ab,kw,ti OR ‘Acute Stroke’:ab,kw,ti OR ‘Acute Strokes’:ab,kw,ti OR ‘Strokes, Acute’:ab,kw,ti OR ‘Cerebrovascular Accident, Acute’:ab,kw,ti OR ‘Acute Cerebrovascular Accident’:ab,kw,ti OR ‘Acute Cerebrovascular Accidents’:ab,kw,ti OR ‘Cerebrovascular Accidents, Acute’:ab,kw,ti |
|  | #2 | ‘Acceptance and Commitment Therapy’:ab,kw,ti OR ‘cognitive behavioral therapy’:ab,kw,ti OR ‘acceptance commitment therapy’:ab,kw,ti OR ‘acceptance commitment treatment’:ab,kw,ti OR ‘acceptance commitment’:ab,kw,ti OR ‘acceptance therapy’:ab,kw,ti OR ‘acceptance treatment’:ab,kw,ti OR ‘commitment therapy”:ab,kw,ti OR commitment treatment’:ab,kw,ti OR ‘ACT’:ab,kw,ti |
|  | #3 | ‘Randomized Controlled Trial’:ab,kw,ti OR ‘randomized’:ab,kw,ti OR ‘placebo’:ab,kw,ti OR ‘RCT’:ab,kw,ti |
|  | #4 | #1 and #2 and #3 |
| CNKI | (SU=(卒中+中风+脑卒中+缺血性脑卒中+脑梗死+腔隙性脑梗死+脑出血+脑血管意外+急性脑梗死) OR TI=(卒中+中风+脑卒中+缺血性脑卒中+脑梗死+腔隙性脑梗死+脑出血+脑血管意外+急性脑梗死) OR AB=(卒中+中风+脑卒中+缺血性脑卒中+脑梗死+腔隙性脑梗死+脑出血+脑血管意外+急性脑梗死) OR KY=(卒中+中风+脑卒中+缺血性脑卒中+脑梗死+腔隙性脑梗死+脑出血+脑血管意外+急性脑梗死)) AND (SU=(接纳承诺疗法+接纳与承诺疗法+承诺与接纳疗法+接纳疗法+承诺疗法+ACT) OR TI=(接纳承诺疗法+接纳与承诺疗法+承诺与接纳疗法+接纳疗法+承诺疗法+ACT) OR AB=(接纳承诺疗法+接纳与承诺疗法+承诺与接纳疗法+接纳疗法+承诺疗法+ACT) OR KY=(接纳承诺疗法+接纳与承诺疗法+承诺与接纳疗法+接纳疗法+承诺疗法+ACT)) AND (SU=(随机对照试验+随机与对照实验+RCT+随机对照研究+随机+随机对照) OR TI=(随机对照试验+随机与对照实验+RCT+随机对照研究+随机+随机对照) OR AB=(随机对照试验+随机与对照实验+RCT+随机对照研究+随机+随机对照) OR KY=(随机对照试验+随机与对照实验+RCT+随机对照研究+随机+随机对照)) | |
| Wanfang | 全部:(卒中 or 中风 or 脑卒中 or 缺血性脑卒中 or 脑梗死 or 腔隙性脑梗死 or 脑出血 or 脑血管意外 or 急性脑梗死) AND 全部:(接纳承诺疗法 or 接纳与承诺疗法 or 承诺与接纳疗法 or 接纳疗法 or 承诺疗法 or ACT) AND 全部:(随机对照试验 or 随机与对照实验 or RCT or 随机对照研究 or 随机 or 随机对照) | |
| VIP | #1 | 任意字段=卒中OR中风OR脑卒中OR缺血性脑卒中OR脑梗死OR腔隙性脑梗死OR脑出血OR脑血管意外OR急性脑梗死 |
|  | #2 | 任意字段=接纳承诺疗法 OR 接纳与承诺疗法 OR 承诺与接纳疗法 OR 接纳疗法 OR 承诺疗法 OR ACT |
|  | #3 | 任意字段=随机对照试验 OR 随机与对照实验 OR RCT OR 随机对照研究 OR 随机 OR 随机对照 |
|  | #4 | #1 and #2 and #3 |
| SinoMed | #1 | "卒中"[不加权:扩展] |
|  | #2 | "中风"[常用字段:智能] OR "脑卒中"[常用字段:智能] OR "缺血性脑卒中"[常用字段:智能] OR "脑梗死"[常用字段:智能] OR "腔隙性脑梗死"[常用字段:智能] OR "脑出血"[常用字段:智能] OR "脑血管意外"[常用字段:智能] OR "急性脑梗死"[常用字段:智能] |
|  | #3 | (#2) OR (#1) |
|  | #4 | 接纳承诺疗法 |
|  | #5 | "接纳与承诺疗法"[常用字段:智能] OR "承诺与接纳疗法"[常用字段:智能] OR "接纳疗法"[常用字段:智能] OR "承诺疗法"[常用字段:智能] OR "ACT"[常用字段:智能] |
|  | #6 | (#5) OR (#4) |
|  | #7 | "随机对照试验"[常用字段:智能] |
|  | #8 | "随机与对照实验"[常用字段:智能] OR "RCT"[常用字段:智能] OR "随机对照研究"[常用字段:智能] OR "随机"[常用字段:智能] OR "随机对照"[常用字段:智能] |
|  | #9 | (#8) OR (#7) |
|  | #10 | (#9) AND (#6) AND (#3) |
